# Mismatch in epitope specificities between IFNγ inflamed and uninflamed conditions leads to escape from T lymphocyte killing in melanoma

**DOI:** 10.1186/s40425-016-0111-7

**Published:** 2016-02-16

**Authors:** Katherine Woods, Ashley J. Knights, Matthew Anaka, Ralf B. Schittenhelm, Anthony W. Purcell, Andreas Behren, Jonathan Cebon

**Affiliations:** School of Cancer Medicine, La Trobe University, Melbourne, VIC 3086 Australia; Olivia Newton-John Cancer Research Institute, Level 5 ONJCWC, 145 Studley Road, Heidelberg, VIC 3084 Australia; Cancer Immunobiology Laboratory, Ludwig Institute for Cancer Research, Melbourne-Austin Branch, Melbourne, Australia; Department of Biochemistry and Molecular Biology, Monash University, Clayton, VIC Australia

**Keywords:** T-cell killing, Antigen processing, Immunoproteasome, Melanoma, Tumor antigens

## Abstract

**Background:**

A current focus in cancer treatment is to broaden responses to immunotherapy. One reason these therapies may prove inadequate is that T lymphocytes fail to recognize the tumor due to differences in immunogenic epitopes presented by the cancer cells under inflammatory or non-inflammatory conditions.

The antigen processing machinery of the cell, the proteasome, cleaves proteins into peptide epitopes for presentation on MHC complexes. Immunoproteasomes in inflammatory melanomas, and in antigen presenting cells of the immune system, are enzymatically different to standard proteasomes expressed by tumors with no inflammation. This corresponds to alterations in protein cleavage between proteasome subtypes, and a disparate repertoire of MHC-presented epitopes.

**Methods:**

We assessed steady state and IFNγ-induced immunoproteasome expression in melanoma cells. Using epitope specific T-lymphocyte clones, we studied processing and presentation of three NY-ESO-1 HLA-Cw3 restricted epitopes by melanoma cell lines. Our experimental model allowed comparison of the processing of three distinct epitopes from a single antigen presented on the same HLA complex. We further investigated processing of these epitopes by direct inhibition, or siRNA mediated knockdown, of the immunoproteasome catalytic subunit LMP7.

**Results:**

Our data demonstrated a profound difference in the way in which immunogenic T-lymphocyte epitopes are presented by melanoma cells under IFNγ inflammatory versus non-inflammatory conditions. These alterations led to significant changes in the ability of T-lymphocytes to recognize and target melanoma cells.

**Conclusions:**

Our results illustrate a little-studied mechanism of immune escape by tumor cells which, with appropriate understanding and treatment, may be reversible. These data have implications for the design of cancer vaccines and adoptive T cell therapies.

**Electronic supplementary material:**

The online version of this article (doi:10.1186/s40425-016-0111-7) contains supplementary material, which is available to authorized users.

## Background

Although immune recognition of cancer occurs frequently, spontaneous immune responses leading to clinical benefit in cancer patients are rare [1, 2]. This failure of effective immunity may result from tumor-mediated immune suppression and evasion. Immunotherapies that counter these mechanisms, such as immune checkpoint inhibition [3, 4] and adoptive transfer of T-lymphocytes [5], have been successfully developed for melanoma and other cancers. These approaches have established that functional T-lymphocytes capable of recognizing specific HLA-peptide complexes on cancer cells can mediate powerful anti-tumor effects. Nevertheless, many patients fail to respond to these therapies, and vaccines designed to generate or enhance specific immunity against tumor antigens have largely been unsuccessful [2]. Further, it is becoming clear that the nature of the tumor microenvironment has a large role to play in success of treatment. For example, in melanoma, the presence of tumor infiltrating lymphocytes (TILs); the frequency of neo-epitopes arising from tumor specific mutations; and the presence of an interferon–gamma related immune signature have all been correlated with positive outcomes for immune checkpoint inhibition [6–8]. Consequently the concept has emerged that a T-lymphocyte/IFNγ inflamed tumor microenvironment carries positive predictive value for responses to immunotherapy.

With regards to the latter, pro-inflammatory cytokines such as IFNγ affect many facets of immunity within the tumor microenvironment, such as the induction of the immune checkpoint molecule PD-L1, cell surface MHC expression, and the configuration of the protein-degrading proteasome complex. These mechanisms can profoundly influence the recognition of tumor antigens by T lymphocytes. Here, we establish the impact of inflammatory conditions on melanoma cells and the consequences of subsequent alterations in the proteasome complex, on T lymphocyte mediated cancer cell recognition.

The degradation of cellular proteins by the proteasome is a critical step in the generation of MHC-associated peptides for presentation to cytotoxic CD8^+^ T lymphocytes [9]. The proteasome is a barrel shaped complex consisting of four stacked rings, each with seven subunits [10]. The outer two rings are composed of structural α-subunits, and the inner two consist of catalytic β subunits. The constitutive ‘standard’ proteasome and the IFN-γ-induced immunoproteasome (IP) differ in their use of three catalytic β subunits. In the immunoproteasome complex, subunits LMP-2, LMP-7 and MECL1 replace the β1, β5 and β2 subunits respectively, resulting in changes in enzymatic activity and production of an altered MHC class I epitope repertoire (reviewed in [11]).

Dendritic cells (DC) constitutively express IP at high levels, [12, 13] whereas tumor cells [14], and specifically melanoma cells [15], are generally described to infrequently express IPs or to express the LMP-2 and −7 subunits at low levels. The potential therefore arises for a different repertoire of epitopes to be produced by DC and tumor cells, and a number of epitopes from melanoma-associated antigens have been described as being differentially processed between the two proteasome subtypes [16]. This phenomenon may be mirrored between tumors with inflammatory versus non-inflammatory microenvironments where epitopes presented by DC for priming of T-lymphocytes will more closely reflect the epitope repertoire of inflammatory tumors.

The cancer testis antigen (CT Ag) NY-ESO-1, and its numerous T-lymphocyte epitopes have been defined and studied in detail [17, 18]. Here, we utilize NY-ESO-1 as a prototypic tumor antigen to investigate how differences in proteasome subtypes can contribute to tumor escape from T lymphocyte recognition. By comparing the processing of three different HLA-Cw3 restricted NY-ESO-1 epitopes; *92*–*100, 96–104* and *124*–*133* [19, 20] we show that inflammation mediated changes in this process can lead to a failure to present appropriate target antigens to cytotoxic T lymphocytes. We demonstrate that optimal processing of each of the NY-ESO-1 epitopes is dependent on different proteasome subtypes, with significant corresponding impact on T-lymphocyte recognition and killing of melanoma cells. Our results comprehensively illustrate the potential for an extraordinary level of disparity in antigen presentation depending on the proteasome subtype present in the cell. Importantly, this study illustrates a mechanism whereby intratumoral inflammation (or lack of it) has the potential to profoundly affect T-lymphocyte killing.

## Results

### Immunoproteasome subunits are differentially expressed in melanoma cell lines

**Fig. 1:**
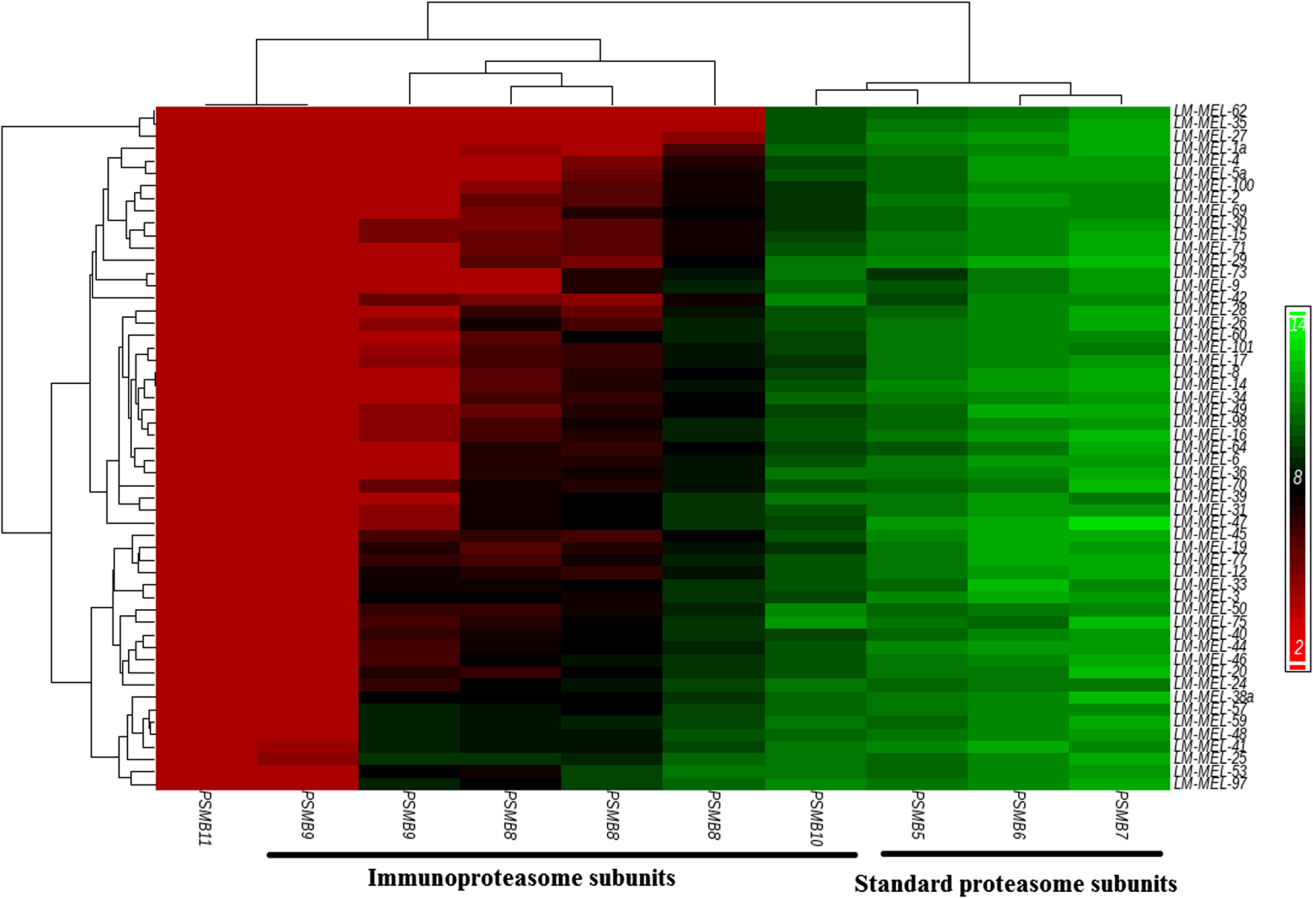
Proteasome subunit expression in melanoma cells lines. Hierarchical clustering of a panel of 55 early passage melanoma cell lines based on expression of standard proteasome subunits *PSMB5, PSMB6, PSMB7*, corresponding immunoproteasome (IP) subunits *PSMB8, PSMB9, PSMB10,* and thymus restricted subunit *PSMB11*

We assessed the mRNA expression levels of standard proteasome subunits (*PSMB5* [Swiss-Prot:P28074]*, PSMB6* [Swiss-Prot:P28074]*, PSMB7* [Swiss-Prot:Q99436]) and immunoproteasome (IP) subunits (*PSMB8* [Swiss-Prot:P28062]*, PSMB9* [Swiss-Prot:P28065]*, PSMB10* [Swiss-Prot:P40306]) in our database of 55 melanoma cell lines by gene expression array analysis [21, 22] (Fig. 1). In line with previous reports, we found that standard proteasome subunits were expressed by all of our cell lines [15]. Interestingly however, the IP subunit *PSMB10* was also expressed by the majority of our cell lines under steady state conditions. Expression of the IP subunit *PSMB8* was variable, detected in some cell lines and not in others, while the third IP-specific subunit, *PSMB9*, was detected in only a few cell lines. The *PSMB11* subunit is a component of the specialized thymus restricted thymoproteasome, and its expression was not detected in any of our cell lines [23].

**Fig. 2:**
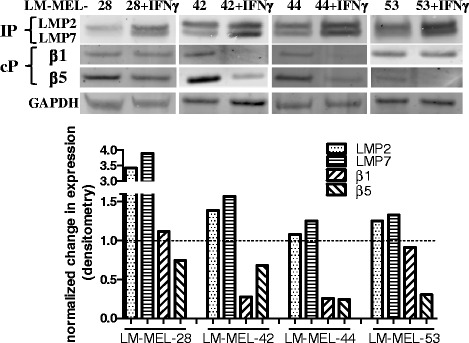
Effect of IFNγ on proteasome subunit expression in melanoma cells lines. Selected melanoma cell lines were incubated +/− IFNγ (100 ng/ml, 72 h) and changes in expression of proteasome subunits was determined by Western blot. (*Top panels*) Expression of the IP subunits LMP2, LMP7 and the standard proteasome subunits β1 and β5 were assessed under steady state and IFNγ treated conditions. (*Bottom panels*) Expression of each subunit was quantified by densitometry analysis using Image Studio software. All blots shown here have been cropped from the original, and some changes to the contrast/brightness have been made for purposes of clarity using Image Studio software

We next evaluated expression of the protein products of constitutive proteasome subunit genes *PSMB5* and *PSMB6* (β5 and β1 respectively), and IP subunit genes *PSMB8* and *PSMB9*, (LMP7, LMP2) by Western blot in 4 selected cell lines. Since expression of the IP is upregulated by IFNγ [24, 25] we assessed proteasome subunit expression both under steady state conditions, and following 72 h incubation with IFNγ (Fig. 2). Even in the absence of IFNγ, some LMP2 and LMP7 expression was observed, which in all cell lines tested was upregulated following incubation with IFNγ, (quantified by densitometry analysis, Fig. 2, bottom panel). A concomitant decrease in expression of the constitutive proteasome subunits β1 and β5 was observed following IFNγ treatment of the same cell lines (Fig. 2).

### Change in proteasome subunit expression alters processing and presentation of NY-ESO-1 epitopes

For the well-studied cancer-testis antigen, NY-ESO-1, three HLA-Cw3 restricted epitopes have been defined; namely, NY-ESO-1_92–100_*,* NY-ESO-1_96–104_ and NY-ESO-1_124–133_ [19, 20]. Using PBMC from melanoma patients we isolated and expanded T-lymphocyte clones which recognized each of these epitopes. The specificity of each T-lymphocyte clone was confirmed by titration against peptide coated PBMC (not shown).

**Fig. 3:**
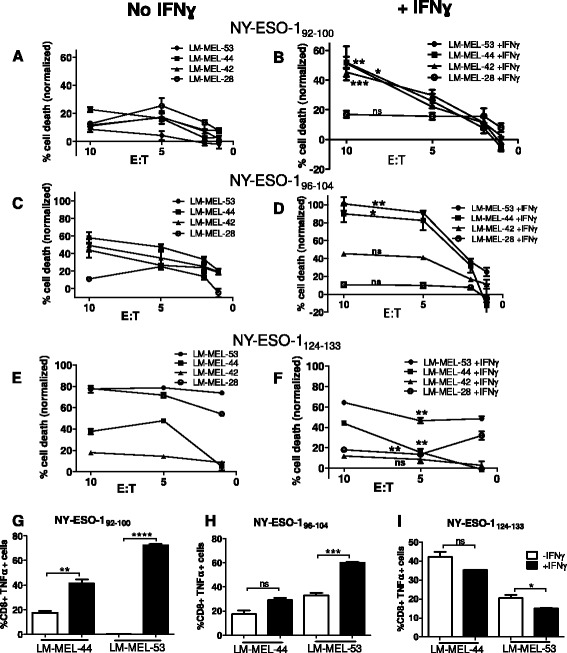
Processing of NY-ESO-1 HLA-Cw3 restricted epitopes by melanoma cells expressing standard or immunoproteasomes. Selected melanoma cell lines were incubated in presence or absence of IFNγ for 72 h to induce expression of the immunoproteasome. Cells were plated in wells of a 96 well flat-bottom plate at 10^4^/well. T-lymphocyte clones specific for NY-ESO-1 HLA-Cw3 restricted epitopes *92*–*100, 96–104*, or *124–133* were added to each cell line at the effector:target (E:T) ratios shown (**a**–**f**) or at 5 × 10^4^/well (**g**, **h**). Cytotoxicity assays, (**a**–**f**): Samples were incubated for 4 (**a**–**d**) or 24 h (**e**, **f**) at 37 °C. T-lymphocyte mediated cytotoxicity was determined by calcein release (**a**–**d**), or by MTS assay (**e**, **f**) and normalized to maximum /spontaneous lysis control wells. Asterisks in the IFNγ treated panels (b, d, f) indicate statistical significance of the change in killing between the equivalent cell line under steady state conditions (a,c,e). Intracellular cytokine stain, (**g**, **h**, **i**): Samples were incubated for 4 h in presence of brefeldin A. T-lymphocytes were incubated with fluorescent antibodies for CD3 and CD8 and stained intracellularly with anti-TNFα antibody, followed by FACS analysis. Error bars represent SEM (*n* = 3). Ns = not significant >0.05; * < 0.05; ** < 0.01; *** < 0.001; **** < 0.0001

To determine the effect of constitutive- versus immuno- proteasome processing on antigen presentation by melanoma cells, we selected HLA-Cw3^+^, NY-ESO-1 expressing cell lines and induced IP expression in each by incubation with IFNγ. We compared subsequent processing and surface presentation of each HLA-Cw3 restricted T-lymphocyte epitope in two ways. Firstly, we determined the ability of specific T-lymphocyte clones to lyse melanoma cells either under steady state conditions (Fig. 3a, c, e), or following induction of an IP (Fig. 3b, d, f). Secondly, we assessed activation of each T-lymphocyte clone in response to melanoma cell lines treated +/− IFNγ, by measuring TNFα secretion (Fig. 3g, h, i). Induction of an IP resulted in significant changes in T-lymphocyte mediated tumor killing or recognition for each of the three epitopes tested, which demonstrated that epitope presentation on the cell surface had altered.

Our results showed that each of the three NY-ESO-1 epitopes tested was processed in a different manner. In most cases T-lymphocytes recognizing the NY-ESO-1_92–100_ and NY-ESO-1_96–104_ epitopes could lyse melanoma cells under steady state conditions (Fig. 3a, c). Of the two epitopes, T-lymphocytes recognizing the NY-ESO-1_96–104_ epitope were generally more effective at baseline killing of these cell lines. However, following induction of an IP in the same cell lines T-lymphocyte mediated tumor lysis was significantly enhanced by NY-ESO-1_92–100_ or NY-ESO-1_96–104_ specific T-lymphocytes in 3/4 and 2/4 cell lines tested respectively (Fig. 3b, d). An increase in killing of up to ~43 % mediated by NY-ESO-1_92–100_ specific T-lymphocytes was observed at a 10:1 E:T ratio (range: 4.3–42.82 %; mean: 23.88 %), and up to ~57 % increased melanoma lysis (range: 0–57.37 %; mean: 19.71 %) mediated by NY-ESO-1_96–104_ specific T-lymphocytes at a 10:1 E:T ratio (Fig. 3b, d). Complementary results were observed for activation of T-lymphocytes recognizing these epitopes on melanoma cell lines treated under the same conditions. In both cases the percentage of activated T-lymphocytes (secreting TNFα) was increased following incubation of melanoma cells with IFNγ (Fig. 3g, h).

In contrast, processing of the NY-ESO-1_124–133_ epitope decreased following induction of the IP in the same cell lines. Specific T-lymphocyte mediated lysis and recognition of this epitope were both reduced significantly in 3/4 cell lines following IFNγ treatment, compared to under steady state conditions when the constitutive proteasome was expressed (Fig. 3e, f, i). Melanoma cell lysis by T-lymphocytes recognizing NY-ESO-1_124–133_ was decreased by up to ~21 % at 10:1 E:T ratio (range: 2.46–21.63 %, mean:12.41 %) when the IP was present.

Since incubation with IFNγ is known to upregulate HLA Class I on the cell surface [26], we sought to confirm that the enhanced T-lymphocyte killing/recognition observed for NY-ESO-1_92–100_ and NY-ESO-1_96–104_ epitopes was not simply due to increased surface HLA. We coated melanoma cells with NY-ESO-1_92–100_ or NY-ESO-1_96–104_ peptides, thereby saturating the HLA molecules, and assessed specific T-lymphocyte cytoxicity (Additional file 1: Figure S1). In comparison with melanoma cells which presented NY-ESO-1 epitopes directly (i.e. were not peptide loaded), cells loaded with either peptide underwent significantly greater lysis mediated by their cognate T-lymphocyte. These results confirm that HLA levels under steady state conditions were not a limiting factor for surface presentation of antigen in these experiments, and ruled out the possibility that an increase in HLA alone would account for an increase in T-lymphocyte mediated cell lysis. Indeed, this was in any case implied by the results showing decreased recognition of the NY-ESO-1_124–133_ epitope in presence of IFNγ.

As a means of directly assessing HLA-presentation of each epitope, we isolated HLA class I complexes by immunoaffinity purification using the mouse monoclonal antibodies DT9 (HLA-C specific) and W6/32 (pan anti-HLA-A,-B,-C) covalently linked to protein A sepharose. The bound peptides were eluted under mild acidic conditions, fractionated by RP-HPLC and analyzed by multiple reaction monitoring (MRM), a targeted mass spectrometry approach which is used to quantify individual peptides in complex mixtures. Despite the unparalleled sensitivity of this technique, we failed to detect presentation of any of these three peptides under either steady state conditions, or following IFNγ treatment (data not shown). This result was disappointing, however not entirely unexpected. Previous studies have highlighted sensitivity of peptide purification by antibody pulldown as a limiting factor of this approach, and it is clear that specific T-lymphocytes have greater sensitivity for their cognate epitope [27].

### Knockdown of PSMB8 by siRNA reverts immunoproteasome mediated processing of NY-ESO-1 HLA-Cw3 restricted epitopes

**Fig. 4:**
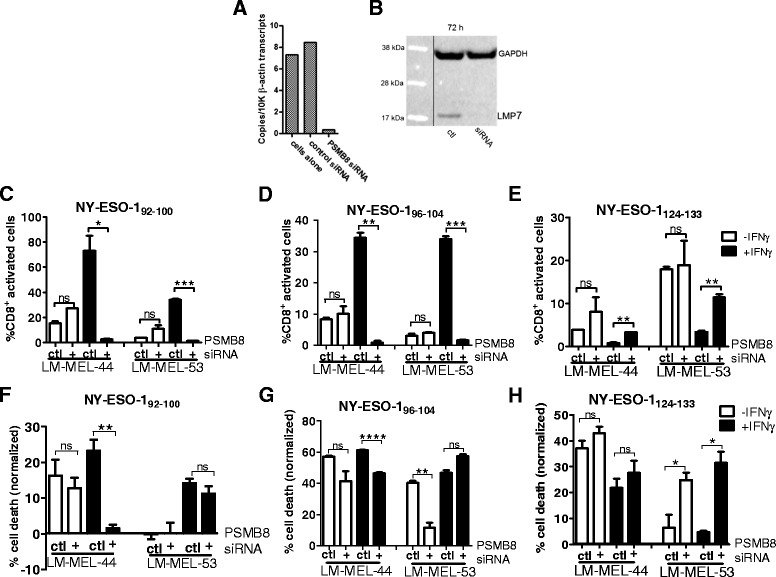
Processing of NY-ESO-1 HLA-Cw3 restricted epitopes following *PSMB8* siRNA knockdown in melanoma cell lines. *PSMB8* expression was knocked down by siRNA in melanoma cell lines and knockdown was confirmed at mRNA (**a**) and protein (**b**) levels following 72 h incubation. Following 72 h of *PSMB8* knockdown alongside incubation +/− IFNγ, T-lymphocyte recognition (**c**–**e**) or cytotoxicity (**f**–**h**) was assessed and compared to cells treated with scrambled control siRNA. (**c**–**e**): T-lymphocytes were incubated with melanoma cells for 4 h in presence of Brefeldin A and cytokine secretion was determined by intracellular staining, followed by FACS analysis. **f**–**h**: T-lymphocytes were incubated with melanoma cells at a 1:1 effector:target ratio for 24 h at 37 °C, then washed off. T-lymphocyte mediated cytotoxicity was determined by MTS assay and normalized to control wells with no T-lymphocytes. Error bars represent SEM (*n* = 3). Ns = not significant >0.05; * < 0.05; ** < 0.01; *** < 0.001

In the process of formation of an IP, the LMP7 subunit is required for processing of other IP specific subunits and assembly of intact IP [28]. LMP7 is also a component of two described intermediate proteasome subtypes [29]. Because these roles of LMP7, we knocked down the expression of the corresponding gene, *PSMB8*, by siRNA (Fig. 4a) and confirmed knockdown at the protein level (Fig. 4b) in melanoma cell lines. These melanoma cells were incubated +/− IFNγ, and the ability of NY-ESO-1 specific HLA-Cw3 restricted T-lymphocytes to recognize or kill melanoma cells lines was assessed (Fig. 4c-h). As previously observed treatment of cell lines with IFNγ resulted in enhanced presentation of the NY-ESO-1_92–100_ and NY-ESO-1_96–104_ epitopes, while presentation of NY-ESO-1_124–133_ decreased (Fig. 4c–h).

The knockdown of LMP7 resulted in complete abrogation of the IFNγ induced enhancement of T-lymphocyte cytotoxicity and recognition of the NY-ESO-1_92–100_ and NY-ESO-1_96–104_ epitopes in most cases (Fig. 4c, d, f, g). For the NY-ESO-1_92–100_ epitope, T-lymphocyte activation following exposure to melanoma cells treated with IFNγ in conjunction with *PSMB8* knock-down, was substantially decreased in comparison to IFNγ together with control siRNA, and indeed was reduced to below that of untreated control cells (Fig. 4c), most likely reflective of knockdown of low level *PSMB8* expression in these cell lines under steady state conditions (Fig. 1). NY-ESO-1_92–100_ specific T-lymphocyte mediated killing was also reverted by concomitant IFNγ treatment and *PSMB8* knockdown (Fig. 4f). In the LM-MEL-44 cell line, this effect was profound, and these T-lymphocytes were effectively unable to lyse this cell line when the LMP7 component of the proteasome was not present. The effect of *PSMB8* knockdown on NY-ESO-1_96–104_ specific T-lymphocyte mediated killing was less profound, however in one cell line specific lysis was significantly decreased following IFNγ/*PSMB8* siRNA treatment compared to cell lines treated with IFNγ and control siRNA (Fig. 4g). Similarly T-lymphocyte recognition of this epitope, enhanced by induction of the IP, was significantly abrogated following *PSMB8* knockdown (Fig. 4d). Processing of the NY-ESO-1_124–133_ epitope was, in contrast, restored significantly following *PSMB8* knockdown, although in most cases did not reach levels equivalent to T-lymphocyte cytotoxicity/recognition prior to IFNγ treatment of these cells (Fig. 4e, h).

### Inhibition of LMP7 reverts immunoproteasome mediated processing of NY-ESO-1 HLA-Cw3 restricted epitopes

The small molecule inhibitor PR-924 (gift from Onyx Pharmaceuticals) selectively inhibits the immunoproteasome subunit LMP7 by specific binding to its active site [30]. The LMP7 enzymatic active site is only exposed in intact immunoproteasomes and use of this inhibitor does not affect the overall LMP7 protein level in the cell (Additional file 2: Figure S2a). We found that our melanoma cells lines had increased sensitivity to PR-924 following co-incubation with IFNγ (Additional file 2: Figure S2b). Nevertheless, the inhibitor was still effective at blocking LMP7 specific enzymatic activity at concentrations where melanoma cells remained viable, which varied depending on the cell line used (Additional file 2: Figure S2c).

**Fig. 5:**
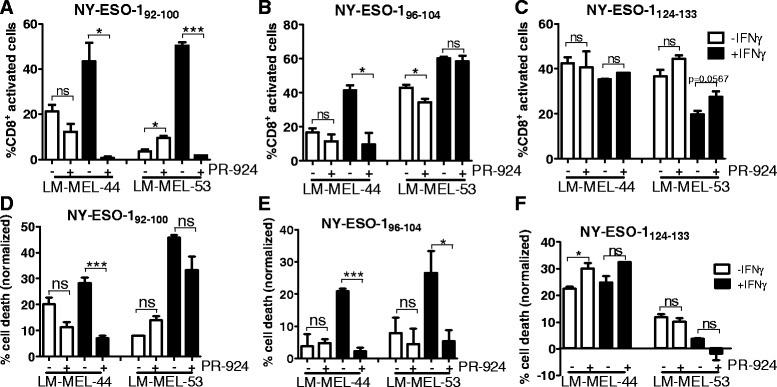
Processing of NY-ESO-1 HLA-Cw3 restricted epitopes following LMP7 inhibition in melanoma cell lines. The enzymatic activity of the immunoproteasome subunit LMP7 was inhibited by incubation of melanoma cell lines with the small molecule inhibitor PR-924, in presence or absence of IFNγ. Following 72 h, T-lymphocytes were added and activation (**a**–**c**) or cytoxicity (**d**–**f**) was assessed for each of the treatment conditions. **a**–**c** T-lymphocytes were incubated with melanoma cells for 4 h in presence of Brefeldin A and cytokine secretion was determined by intracellular staining, followed by FACS analysis. **d**–**f** T-lymphocytes were incubated with melanoma cells at a 1:1 effector:target ratio for 24 h at 37 °C, then washed off. T-lymphocyte mediated cytotoxicity was determined by MTS assay and normalized to control wells with no T-lymphocytes. Error bars represent SEM (*n* = 3). ns = not significant >0.05; * < 0.05; *** < 0.001

Melanoma cell lines were incubated with PR-924 in presence/absence of IFNγ for 72 h and the ability of NY-ESO-1 specific HLA-Cw3 restricted T-lymphocytes to recognize or kill melanoma cells lines was assessed (Fig. 5). As previously observed enhanced presentation of the NY-ESO-1_92–100_ and NY-ESO-1_96–104_ epitopes, and decreased processing of the NY-ESO-1_124–133_ epitope occurred following IFNγ treatment.

NY-ESO-1_92–100_ specific T-lymphocyte recognition/cytotoxicity was generally not significantly affected following incubation with PR-924 alone (Fig. 5a, d). When melanoma cells were incubated in presence of both PR-924 and IFNγ, T-lymphocyte responses were significantly reduced compared to with melanoma cells treated with IFNγ alone, and reverted to activation/cytoxicity levels comparable with, or even less than, those observed for cells treated with PR-924 alone in most cases.

For the NY-ESO-1_96–104_ epitope, incubation of melanoma cell lines with PR-924 alone had generally little effect on T-lymphocyte recognition or cytotoxicity, however co-incubation with PR-924 and IFNγ resulted in decreased T-lymphocyte responses compared to IFNγ alone, which in most cases reverted to levels comparable with standard proteasome mediated processing of this epitope (Fig. 5b, e).

In some cases processing of the NY-ESO-1_124–133_ epitope was slightly enhanced following incubation with PR-924 alone, most likely corresponding to inhibition of minimal immunoproteasome subunits present under steady state conditions (Fig. 5c, d). Treatment of melanoma cells with both PR-924 and IFNγ also slightly enhanced T-lymphocyte responses to the NY-ESO-1_124–133_, however not significantly or to the levels observed under steady state conditions.

### Immunoproteasome expression is correlated with T lymphocyte infiltration in human biopsies

**Fig. 6:**
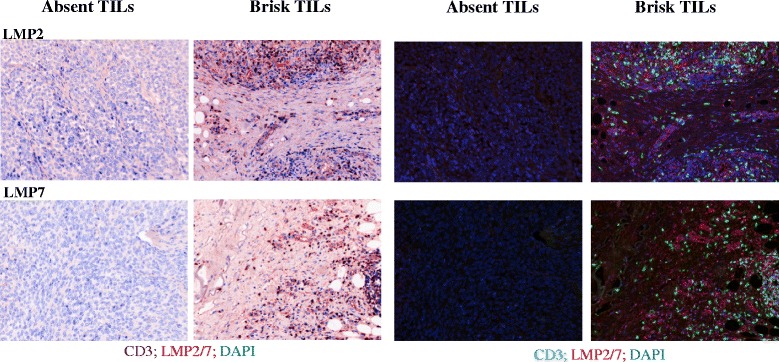
Immunoproteasome expression in melanoma biopsies. Formalin fixed paraffin embedded human melanoma biopsies from a single patient were scored by a pathologist as having ‘absent’ or ‘brisk’ tumor infiltrating lymphocytes (TILs) present, as labeled. 4 μM sections were co-stained for expression of CD3 to identify TILs, and either of the immunoproteasome subunits LMP2 or LMP7 as indicated. DAPI was used to visualize cell nuclei. Images were acquired using a VECTRA automated quantitative pathology imaging system and analysis was performed using InForm software. Images are shown as pseudo-brightfield IHC (*left panels*), or multifocal fluorescent images (*right panels*)

Biopsies from melanoma patients were evaluated by an independent pathologist for the presence of tumor infiltrating lymphocytes (TILs). TILs were scored as being either ‘absent’ or ‘brisk’ [31]. Tumors with brisk TIL infiltrates were deemed to demonstrate inflamed conditions with a likelihood of corresponding IFNγ expression from inflammatory cells [7, 8]. In order to evaluate the IP expression biopsies were selected from individual patients who had more than one metastasis and where paired metastases from these individuals were disparate for TIL. Formalin fixed paraffin embedded (FFPE) tumor sections were stained for the expression of the IP (either LMP2 or LMP7) and presence of CD3^+^ TILs. In 3/3 patients tested, we found that when TILs were absent, IP expression was not detectable. In contrast, both LMP2 and LMP7 were consistently expressed in the presence of TILs, (Fig. 6, representative donor). We found that in sections from a single biopsy, regions which lacked TILs also lacked IP subunit expression, i.e. IP expression was not uniform throughout the ‘inflamed’ tumor. Instead, there was a clear correlation in expression of IP subunits and CD3^+^ cells.

## Discussion

The immune recognition of cancer antigens following vaccination may fail because APC and cancers can generate antigens using different processing machinery. Previous reports have shown that differences in proteasome form can alter processing of individual peptides [32]. Here we have extended those studies by comparing three epitopes from a single tumor antigen (NY-ESO-1) which are presented by the same HLA complex (HLA-Cw3). Our results clearly demonstrate that a mismatch occurs between the specificities of epitopes that were generated between tumor cells under inflammatory/non-inflammatory conditions. This is the first demonstration that processing of a number of epitopes derived from the same tumor antigen can vary depending on the proteasome subtype and subsequent processing of the antigen. Of particular interest is the observation that processing of one epitope (NY-ESO-1_124–133_) was decreased via the IP. There is precedence for decreased processing of individual immunogenic epitopes via the IP (e.g. Melan A_26–35_ [13]), however this has not been previously shown to occur asynchronously with other epitopes from the same protein.

We demonstrated decreased processing in 4/4 NY-ESO-1^+^, HLA-Cw3^+^ cell lines (Fig. 3e, f), despite variability of these cell lines in their IP subunit expression (Fig. 2). The IP knockdown and inhibition experiments confirmed the dependence of NY-ESO-1_124–133_ on processing via the standard proteasome. In these experiments, NY-ESO-1_124–133_ processing in presence of IFNγ could be partially or completely rescued by knockdown/inhibition of PSMB8, since under these conditions only standard proteasomes can be formed (siRNA) (Fig. 4e, h), or are enzymatically active (Fig. 5c, f).

Given these findings it seems likely that a mismatch between vaccine-generated and tumor-displayed epitopes may occur with greater frequency than has been previously appreciated, particularly if the tumor micro-environment is uninflamed. This concept is supported by the work of Saccheri et al. [33]. This group infected melanoma cells with bacteria which facilitated the formation of gap junctions between melanoma cells and DC, and the subsequent direct transfer of melanoma processed (via the standard proteasome) epitopes onto DC. These DC were capable of inducing anti-tumor immunity which led to a decrease in tumor growth. DC incubated with tumor lysate, necessitating cross presentation of antigen via the immunoproteasome, were less efficient at inducing anti-tumor immunity.

Further studies will be required to establish the extent to which this contributes to the failure of cancer vaccines, nonetheless such observations become critical when designing antigen-specific therapies, particularly cancer vaccines and adoptive T-lymphocyte transfer since the fine specificities of peptide antigens on target cells will need to be taken into account.

We have performed a series of clinical trials where patients were vaccinated with full-length NY-ESO-1 recombinant protein (NCT00199901, NCT00518206) ([34] and manuscript in preparation). They were undertaken on the background of detailed studies of NY-ESO-1 epitopes and specific T cell responses [35–37]. These studies have demonstrated that the vaccine is effective at generating diverse immune responses to many epitopes restricted by a variety of HLA alleles [18, 38, 39]. Among these, we have characterized responses to the NY-ESO-1_92–100_ and NY-ESO-1_96–104_, epitopes which were generated in response to vaccination [34]. In contrast, NY-ESO-1_124–133_ specific T-lymphocytes were found to occur spontaneously but were not induced by vaccination. The clinical outcome of the most recent phase II randomized clinical trial will be reported in due course, however suffice to say, like many other reported vaccine trials, it had little impact on patient survival. While there are other mechanisms that can account for this outcome, the findings described here highlight the possibility that a dissociation between the peptides generated by APC from those present on tumor may indeed be an obstacle to effective cancer recognition. This possibility is further supported by a recent clinical trial reported by Dannull et al. showing that melanoma patients vaccinated with tumor antigen loaded DC, which had been co-transfected with siRNA targeting the three IP subunits, developed T-lymphocyte responses with superior lytic ability against autologous tumor compared to control siRNA transfected DC [40, 41].

The presence of IFNγ within the tumor microenvironment has been shown to be associated with improved clinical outcomes to both cancer vaccination and immune checkpoint inhibition [7]. While IFNγ could influence immune recognition at a variety of levels, one now obvious effect could be by correcting the disparity in epitope repertoires between those displayed by APC and tumor. Indeed, Donia et al. demonstrated that tumor cells treated with IFNγ ex vivo were recognized better by autologous TILs [42]. Our results clearly demonstrate that upregulation of the IP occurs in the T lymphocyte inflamed tumor microenvironment in vivo (Fig. 6)*.* It is tempting to speculate that the presence of TILs secreting IFNγ leads directly to IP upregulation. Further study is underway in our lab to further define the mechanism of the in vivo upregulation of the IP, as well as to correlate this with patient prognosis and/or response to treatment. Immune checkpoint inhibitors are now routinely used in treatment of melanoma, and these antibodies have the capacity to induce IFNγ at the tumor site [43, 44]. Based on our results, other agents that induce IFNγ at the tumor site may also facilitate enhanced tumor recognition by T-lymphocytes that have been previously primed by DC to recognize IP generated epitopes. Our ongoing laboratory studies are addressing this in pre-clinical models.

Since IFNγ upregulates PD-L1 expression by melanoma cell lines, [45] (Additional file 3: Figure S3 a,b) we assessed whether concomitant treatment of melanoma cells with IFNγ and PD-1 inhibitory antibody resulted in further enhancement of T-lymphocyte lysis by T-lymphocyte clones specific for the IP processed the NY-ESO-1_92–100_ epitope (Additional file 3: Figure S3c, d). We demonstrated an additive effect for IP induction and PD-1 inhibition when an appropriate T-lymphocyte epitope was selected as a target.

Shared antigens such as NY-ESO-1 represent attractive targets for immunotherapy, since they are widely expressed in many cancer types and restricted to cancer. Vaccines provide the potential to induce immune effectors that can target cancer, an approach that is particularly attractive when immune ignorance is an obstacle to effective cancer immunotherapy. Based on our studies, however, vaccination alone may well be ineffective even if effectors are generated, since they may fail to adequately recognize their targets. This problem may be readily overcome with a further refinement; the addition of strategies that generate IFNγ in the tumor micro-environment and induce IP expression. There are many ways this could be achieved including antibody targeting [46], oncolytic viruses [47], and the use of kinase inhibitors. Such approaches clearly warrant clinical evaluation, particularly in patient populations for whom existing immunotherapies fail to have impact.

## Conclusions

We found that differences in proteasomes between inflamed and uninflamed tumors led to profound changes in the generation of three distinct epitopes from a single tumor antigen. The observed changes in epitope repertoire between inflamed and uninflamed tumors resulted in significant differences in the ability of T lymphocytes to target tumor cells. Our results demonstrate that knowledge of the tumor microenvironment, and how to manipulate it, may be critical for the success of cancer vaccine or adoptive T cell therapies and that such therapies may be more effective when combined with strategies to induce inflammation at the tumor site.

## Methods

### Human ethics approval

Samples used in this study were derived from patients who consented to participate in a clinical research protocol approved by Austin Health Human Research Ethics Committee (HREC H2006/02633).

### Melanoma cell line culture

Establishment and characterization of the melanoma cell lines used has been previously described [21, 22]. Cells were cultured in RF10 consisting of RPMI 1640, 2 mM Glutamax, 100 IU/ml Penicillin, 100 μg/ml Streptomycin and 10 % heat-inactivated fetal calf serum (all Invitrogen). For induction of immunoproteasome catalytic subunits cells were incubated with 100 ng/ml IFNγ (Peprotech) for 72 h prior to experiments. For inhibition of PD-1, anti-PD-1 antibody (nivolumab, BMS) was added at 10 μg/ml alongside the T-lymphocyte clones.

### Gene expression array

The gene expression array dataset [22] and pre-processing methods [48] have been previously described. Briefly, RNA was purified from melanoma cell lines (Qiagen AllPrep kits) and assayed using Illumina HT12 arrays following the manufacturer’s protocols. Hierarchical clustering was performed in Partek Genomic Suite using Euclidian distance and average linkage.

### Western blot

Melanoma cell line lysates were subjected to standard Western blot using NuPAGE® SDS-PAGE pre-cast gels (Invitrogen), and SeeBlue® Plus2 pre-stained protein standards (Invitrogen). Primary antibodies and dilutions were: rabbit anti-human LMP7 (1:1000), LMP2 (1:1000), β5 (1:1000), β1 (1:500), GAPDH (1:2000), (all Abcam). Dilutions were made in standard Tris Buffered Saline with 5 % Bovine serum albumin and 0.05 % Tween20. The secondary antibody was goat anti-rabbit IRDye 800 used at 1:20,000 dilutions. Antibody binding was visualized using an Odyssey Imaging system and ImageStudio Lite Ver 5.2 analysis software (LI-COR).

### Generation and culture of NY-ESO-1 specific CD8^+^ T-lymphocyte clones

CD8^+^ T-lymphocyte clones specific for the NY-ESO-1 HLA-Cw3-restricted peptides NY-ESO-1_92–100_, NY-ESO-1_96–104_, and NY-ESO-1_124–133_ were generated from patients who consented to participate in a clinical research protocol approved by Austin Health Human Research Ethics Committee (HREC H2006/02633). PBMC were stimulated with 1 × 10^−6^ M peptide (Mimotopes), and cultured for 10 days in the presence of 25 IU/ml IL-2 (Peprotech). Specific cells were labeled with a fluorescent tetramer (comprising the relevant peptide and HLA, (TCMetrix, Epalinges, Switzerland) and single-cell sorted using a MoFlow cytometer. Clones were expanded with pooled, autologous healthy donor PBMC as feeder cells, 1 μg/ml phytohaemagglutinin-L (PHA-L (Sigma)) and 600 IU/ml IL-2 (Cetus). After approximately 20 days, 1–10 × 10^3^ clones were restimulated in the presence of autologous PBMC as feeder cells, PHA-L and IL-2, as described above. Clone specificity was confirmed by tetramer staining.

T-lymphocyte clones/lines were cultured in RPMI 1640 media supplemented with 2 mM Glutamax, 100 IU/ml Penicillin, 100 μg/ml Streptomycin, 20 mM HEPES, 1 % nonessential amino acids, 1 mM sodium pyruvate, 55 μM β-mercaptoethanol, and 10 % human serum (TCRPMI). IL-2 (100 IU/ml) was added and replaced every 3 days.

### T-lymphocyte killing – Calcein release assay

Measurement of calcein release from labeled cells as a means of determining T-lymphocyte mediated cytotoxicity has been described previously [49]. Briefly, melanoma cells were stained with 15 μM Calcein-AM reagent (Invitrogen) for 30 min at 37 °C, then washed and plated in a 96-well plate at 10,000 cells/well. T-lymphocytes were added to selected wells as appropriate to give the effector to target (E:T) ratios shown in the text. Samples were incubated for 4 h at 37 °C. Supernatants from each well were transferred to a new 96-well plate, and fluorescence was measured using the Wallac plate reader (excitation 485 nm/emission 535 nm; 0.1 s). Percent cytotoxicity was calculated using the formula: 100 × ([test value − spontaneous]/[maximum − spontaneous]), where ‘spontaneous’ is the measurement from untreated control wells, and ‘maximum’ the reading from cells lysed with 3 % Triton X-100 for 10 min at 37 °C.

### T-lymphocyte killing – MTS assay

Twenty thousand melanoma cells were plated in wells of a flat bottom 96-well plate. T-lymphocytes were added to selected wells as appropriate to give the effector to target (E:T) ratios shown in the text/figure legends. Samples were incubated overnight (~16–24 h) at 37 °C. The following day T-lymphocytes were resuspended by gentle pipetting and then removed. The plate was washed once with PBS. MTS reagent (CellTiter 96® AQueous One Solution Cell, Promega) was added and samples incubated for 1–2 h at 37 °C. Absorbance at 490 nm was measured, and normalized to melanoma samples incubated in absence of T-lymphocytes for each cell line to give percentage of viable cells.

### Intracellular cytokine staining (ICS) of antigen-activated T-lymphocytes

Specific T-lymphocyte clones were incubated for 4 h with melanoma cells in presence of 10 μg/ml brefeldin A. Cells were labeled with live/dead fixable violet stain (Invitrogen) according to the manufacturer’s instructions, then incubated with antibodies against CD3 (PE) and CD8 (APC) (BD biosciences) for 15 min at 4 °C. Samples were washed and fixed with fix/permeabilisation reagent (BD biosciences) for 20 min at 4 °C. Cells were stained with anti-IFNγ-FITC and anti-TNFα-PECy7 (eBiosciences) in permeabilisation/wash solution (BD biosciences) for 25 min at 4 °C. The gating strategy was: SSC/LD^−^; CD3^+^/CD8^+^; CD8^+^/IFNγ^+^ or CD8^+^/TNFα^+^. Data from at least 100,000 stained cells were acquired on a FACSCanto and analyzed with FlowJo software. Data collection and analysis was in accordance with the MIATA guidelines [50].

### Flow cytometry

For PD-L1 analysis, melanoma cells were incubated with anti-CD274-PE (BD bioscience) at 1:50 dilution for 15 min at 4 °C.

Cells were washed twice in PBS then data from at least 100,000 stained cells were acquired on a FACSCanto and analyzed with FlowJo software. Data collection and analysis was in accordance with the MIATA guidelines [50].

### Quantitative Real-Time Polymerase Chain Reaction (qRT-PCR)

cDNA was generated using the High Capacity cDNA Reverse Transcription Kit (Applied Biosystems). Quantitative polymerase chain reaction (qPCR) was performed using the QuantiFast SYBR Green PCR Kit (Qiagen). Primer sequences were as follows: NY-ESO-1, (forward) 5′-gagccgcctgcttgagtt-3′ and (reverse) 5′-agcactgcgtgatccacatc-3′; PSMB8 (forward) 5′-catgggccatctcaatctg-3′, and (reverse) 5′-agtagatttccgcgtcgaag-3′; β-actin (forward) 5′-ctggaacggtgaaggtgaca-3′ and (reverse) 5′-cggccacattgtgaactttg-3′

### PR-924

PR-924 is an epoxyketone which selectively and irreversibly inhibits the LMP7 subunit [30, 51] (Onyx Pharmaceuticals, an Amgen subsidiary, kind gift). Inhibition of LMP7 activity and viability varied between cell lines, and was optimized for each cell line individually as discussed in the text and shown in Additional file 2: Figure S2.

### Chymotrypsin activity assay

Chymotryspin activity in melanoma cells was measured using the Proteasome-Glo™ Chymotrypsin-like cell based assay kit (Promega) according to the manufacturer’s instructions.

### siRNA

siRNA mediated interference was performed using ON-TARGETplus SMARTpool siRNA reagents (Dharmacon) for PSMB8, or scrambled control. Transfections were performed using the RNAiMAX reagent (Life Technologies) and according to the manufacturer’s instructions. Melanoma cell lines were plated at ~60 % confluency and siRNA reagents added at a final concentration of 10 nM. Cells were incubated for 72 h at 37 °C prior to use in experiments.

### Purification of HLA bound peptides

HLA bound peptides were isolated by immunoaffinity chromatography and reverse-phase high-performance liquid chromatography (RP-HPLC) essentially as described before [52, 53]. In brief, 10^9^ cells were lysed in 0.5 % IGEPAL, 50 mM Tris–HCl pH 8.0, 150 mM NaCl and protease inhibitors (CompleteProtease Inhibitor Cocktail Tablet; Roche Molecular Biochemicals) for 45 min at 4 °C. The lysate was cleared by ultracentrifugation at 40,000 g and the HLA class I complexes were immunoaffinity purified using a combination of DT9 (HLA-C specific) and W6/32 monoclonal antibody (pan anti-HLA-A, B, C) covalently linked to protein A sepharose (GE Healthcare). The bound peptides were eluted by acidification with 10 % acetic acid and separated from the class I heavy chains and ß2m molecules on a 50 mm monolithic C18 RP-HPLC column (Chromolith Speed Rod; Merck) using an EttanLC HPLC system (GE Healthcare).

### Multiple Reaction Monitoring (MRM)

Skyline v2.1.0.4936 [54] was used to design MRM transitions for the NY-ESO-1 HLA-Cw3-restricted peptides NY-ESO-1_92–100_, NY-ESO-1_96–104_, and NY-ESO-1_124–133_. For each peptide, the four most abundant b- and y-fragment ions of the predominant precursor ion were selected based on the peptide’s fragmentation pattern on a 5600^+^ TripleTOF mass spectrometer (AB SCIEX). For MRM detection, a QTRAP 5500 mass spectrometer (AB SCIEX) coupled to a NanoUltra cHiPLC system (Eksigent) was used as described previously [55].

### Immunohistochemistry

4 μM sections were cut from formalin fixed paraffin embedded (FFPE) biopsy blocks from selected melanoma patients, and mounted on glass slides. Samples were dewaxed using xylene, then dehydrated by sequential incubation in 100 %, 70 % ethanol, followed by hydrogen peroxide.

Antigen was retrieved by boiling for 15 min in citrate buffer, pH 6.0 (Thermo Scientific). Following cooling/washing with TBST (Thermofisher), slides were blocked by 30 min incubation with Background Sniper reagent (Biocare medical). Slides were then incubated for 1 h with the first primary antibody, either rabbit-anti human LMP2 (Abcam, ab3328) or rabbit-anti-human LMP7 (Abcam, ab3329). Both were used at 0.1 μg/ml diluted in antibody diluent (DAKO). Anti-rabbit-HRP-polymer (DAKO) was added for 30 min, followed by a 1/50 dilution of OPAL-570 TSA in amplification diluent (both from the OPAL 4-color fluorescent IHC kit, Perkin Elmer) for 10 min.

Samples were boiled for 3 min in citrate buffer, pH 6.0 and the above steps were repeated with the second primary antibody, rabbit anti-human CD3 (Abcam, SP7), used at 1/1500 dilution. The only difference in this round of staining was that OPAL-520 TSA (1/50) was used as the fluorophore.

Samples were boiled in citrate buffer, pH 6.0, then stained with DAPI (Life Technologies) diluted 1/2000 in water, for 5 min at room temperature. Coverslips were mounted to slides using Vectashield HardSet Mounting medium (Abacus).

## Additional files

Additional file 1: Figure S1. T-lymphocyte mediated lysis of melanoma cell lines is not limited by HLA. Melanoma cell lines were either untreated, or pulsed with *92*–*100* or *96*–*104* peptide (1 μM/ml for 2 h at room temperature). T-lymphocytes recognising the relevant epitope were incubated with melanoma cells (peptide loaded (right panels) or not (left panels)) and following overnight incubation, percentage cytotoxicity was determined by calcein assay. Results demonstrate that T-lymphocyte mediated cytotoxity of melanoma cells is not limited by HLA, but rather by levels of antigen present on the cell surface. (PDF 11 kb) Additional file 2: Figure S2. Effect of PR-924 on melanoma cell viability and enzymatic activity. (**A**) Since PR-924 inhibits only intact, enzymatically active immunoproteasomes, expression of LMP7 (target of PR-924) was detected by Western blot before and after induction with IFNγ, and in the presence of PR-924 for 72 h. (**B**) Melanoma cells were incubated for 72 h with a dose range of PR-924 as shown, in presence or absence of IFNγ. Viability was determined by MTS assay. IC50 was variable between cell lines, and was reduced following IFNγ incubation in all cases. Error bars represent SEM. (**C**) The LMP7 subunit has chymotrypsin-like enzymatic activity. PR-924 activity was confirmed in melanoma cell lines treated with IFNγ, using a chymotrypsin-like assay (Promega) for enzymatic activity of LMP7 at different doses as indicated. (PDF 65 kb) Additional file 3: Figure S3. Effect of PD-1 inhibition on T-lymphocyte lysis of IFNγ treated melanoma cell lines. Melanoma cell lines were incubated in presence or absence of IFNγ for 72 h at 37 °C. (**A**, **B**) Cells were stained with anti-CD-274 (PD-L1)-APC antibody and the percentage PD-L1 positive melanoma cells was determined by FACS analysis. (**C**, **D**). Melanoma cells, pre-treated +/− IFNγ for 72 h were incubated with T-lymphocyte clones specific for NY-ESO-1_*92–100*_ *,* at 1:1 effector:target ratio, for 24 h at 37 °C, in presence or absence of anti-PD-1 inhibitory antibody (10 μg/ml). T-lymphocytes were washed off following the incubation period and T-lymphocyte mediated cytotoxicity was determined by MTS assay, normalized to control wells with no T-lymphocytes. Error bars represent SEM (n = 3). ns = not significant >0.05; * < 0.05; ** < 0.01. (PDF 12 kb)

## Abbreviations

APC: allophycocyanin
BFA: brefeldin A
CT Ag: cancer testis antigen
DC: dendritic cells
E:T: effector:target
FFPE: formalin fixed paraffin embedded
FITC: fluorescein isothiocyanate
HLA: human leukocyte antigen
HREC: Human Research Ethics Committee
ICS: intracellular cytokine stain
IFNγ: interferon gamma
IHC: immunohistochemistry
IL-2: interleukin 2
IP: immunoproteasome
LMP2: low molecular weight protein 2
LMP7: low molecular weight protein 7
MecL1: multicatalytic endopeptidase complex-like 1
MHC: major histocompatibility complex
MIATA: minimal information about T cell assays
MRM: multiple reaction monitoring
MTS: 3--5--2--2H-tetrazolium
NY-ESO-1: New York Esophageal squamous cell carcinoma-1
PBMC: peripheral blood mononuclear cells
PBS: phosphate buffered saline
PD-1: programmed cell death-1
PD-L1: programmed cell death-ligand 1
PE: phycoerythrin
PECy7: phycoerythrin with cyanin-7
PHA-L: phytohaemagglutinin-L
qPCR: quantitative PCR
RP-HPLC: reverse-phase high performance liquid chromatography
RPM1: Roswell Park Memorial Institute
siRNA: small interfering ribonucleic acid
TBST: Tris buffered saline with Tween-20
TILs: tumor infiltrating lymphocytes
TNFα: tumor necrosis factor alpha
