# Supplementary figures and images for: Mismatch in epitope specificities between IFNγ inflamed and uninflamed conditions leads to escape from T lymphocyte killing in melanoma
Source: J Immunother Cancer. 2016 Feb 16;4:10. doi: 10.1186/s40425-016-0111-7 (PMC4754849; doi:10.1186/s40425-016-0111-7)

## Direct presentation

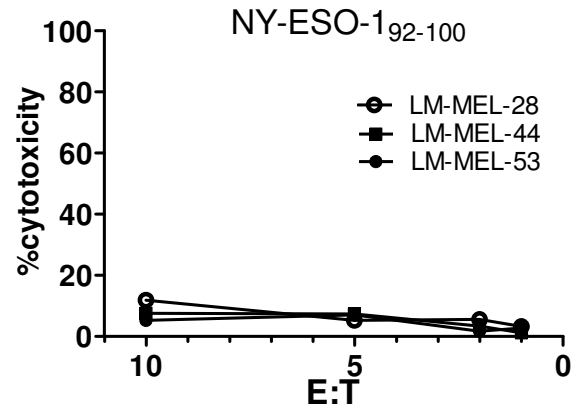

## Peptide pulsed

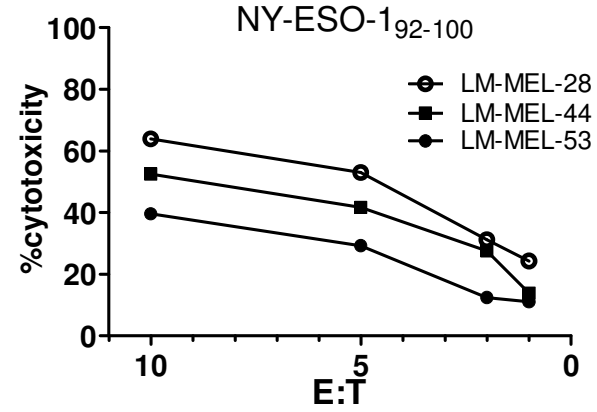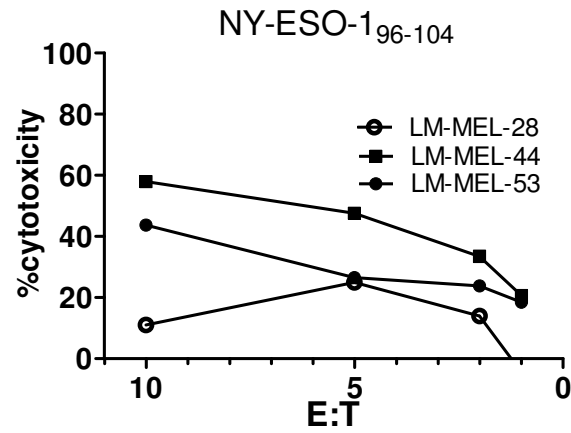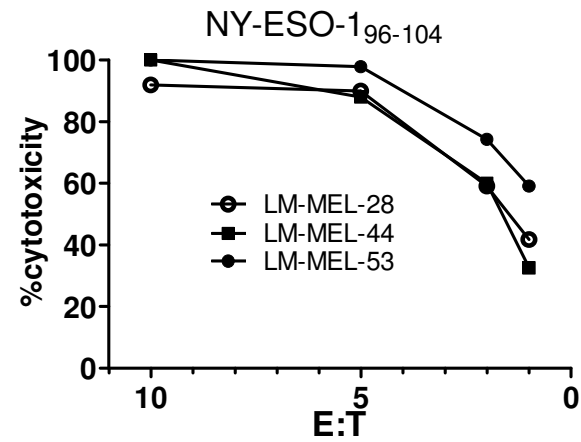

Supplement: Additional file 1: Figure S1. — T-lymphocyte mediated lysis of melanoma cell lines is not limited by HLA. Melanoma cell lines were either untreated, or pulsed with 92–100 or 96–104 peptide (1 μM/ml for 2 h at room temperature). T-lymphocytes recognising the relevant epitope were incubated with melanoma cells (peptide loaded (right panels) or not (left panels)) and following overnight incubation, percentage cytotoxicity was determined by calcein assay. Results demonstrate that T-lymphocyte mediated cytotoxity of melanoma cells is not limited by HLA, but rather by levels of antigen present on the cell surface. (PDF 11 kb) [file 40425_2016_111_MOESM1_ESM.pdf]

**A** LM-MEL-44

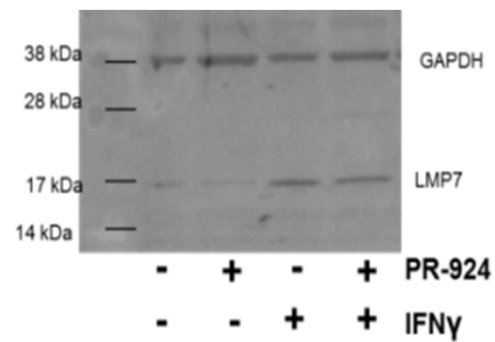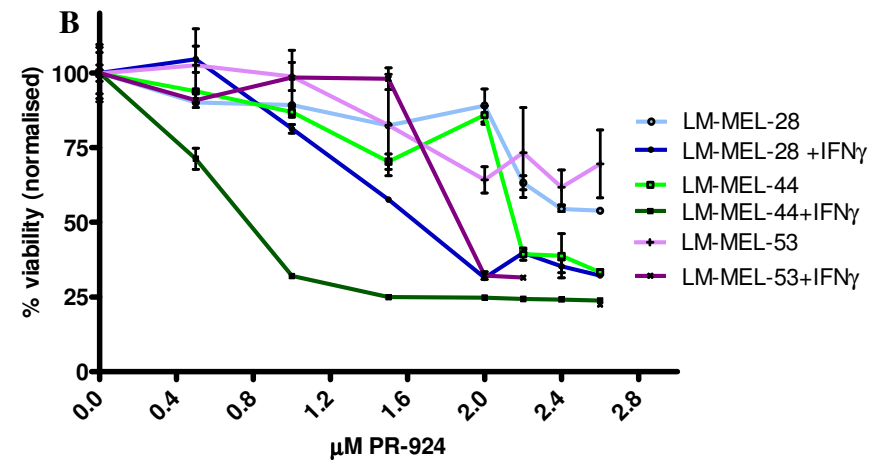

**C**

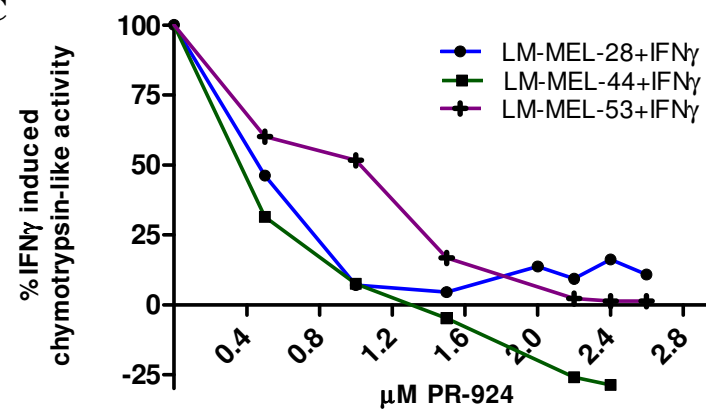

Supplement: Additional file 2: Figure S2. — Effect of PR-924 on melanoma cell viability and enzymatic activity. (A) Since PR-924 inhibits only intact, enzymatically active immunoproteasomes, expression of LMP7 (target of PR-924) was detected by Western blot before and after induction with IFNγ, and in the presence of PR-924 for 72 h. (B) Melanoma cells were incubated for 72 h with a dose range of PR-924 as shown, in presence or absence of IFNγ. Viability was determined by MTS assay. IC50 was variable between cell lines, and was reduced following IFNγ incubation in all cases. Error bars represent SEM. (C) The LMP7 subunit has chymotrypsin-like enzymatic activity. PR-924 activity was confirmed in melanoma cell lines treated with IFNγ, using a chymotrypsin-like assay (Promega) for enzymatic activity of LMP7 at different doses as indicated. (PDF 65 kb) [file 40425_2016_111_MOESM2_ESM.pdf]

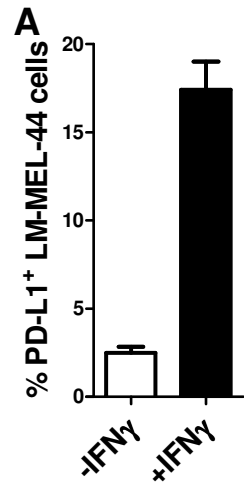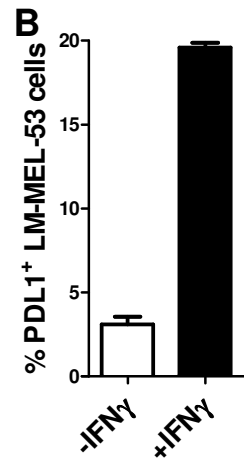

### LM-MEL-44

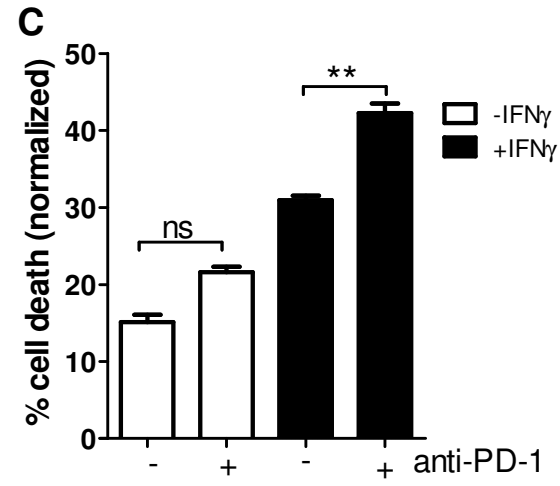

### LM-MEL-53

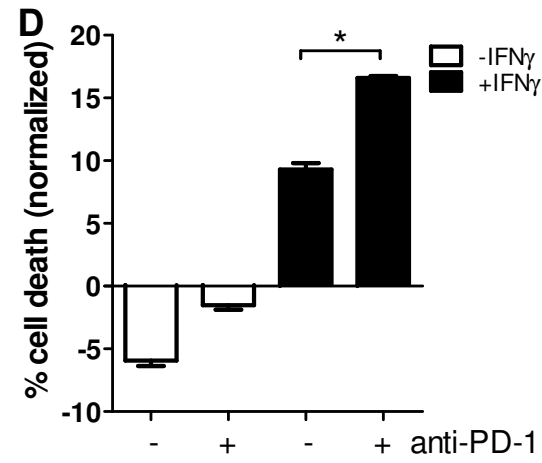

Supplement: Additional file 3: Figure S3. — Effect of PD-1 inhibition on T-lymphocyte lysis of IFNγ treated melanoma cell lines. Melanoma cell lines were incubated in presence or absence of IFNγ for 72 h at 37 °C. (A, B) Cells were stained with anti-CD-274 (PD-L1)-APC antibody and the percentage PD-L1 positive melanoma cells was determined by FACS analysis. (C, D). Melanoma cells, pre-treated +/− IFNγ for 72 h were incubated with T-lymphocyte clones specific for NY-ESO-192–100 , at 1:1 effector:target ratio, for 24 h at 37 °C, in presence or absence of anti-PD-1 inhibitory antibody (10 μg/ml). T-lymphocytes were washed off following the incubation period and T-lymphocyte mediated cytotoxicity was determined by MTS assay, normalized to control wells with no T-lymphocytes. Error bars represent SEM (n = 3). ns = not significant >0.05; * < 0.05; ** < 0.01. (PDF 12 kb) [file 40425_2016_111_MOESM3_ESM.pdf]
